# Beyond the 95s: What happens when uniform program targets are applied across a heterogenous HIV epidemic in Eastern and Southern Africa?

**DOI:** 10.1371/journal.pgph.0003723

**Published:** 2024-09-19

**Authors:** Rachael H. Joseph, Yaa Obeng-Aduasare, Thomas Achia, Abraham Agedew, Sasi Jonnalagadda, Abraham Katana, Elijah J. Odoyo, Aoko Appolonia, Elliot Raizes, Amy Dubois, John Blandford, Lucy Nganga

**Affiliations:** 1 Division of Global HIV & TB, Global Health Center, U.S. Centers for Disease Control and Prevention, Pretoria, South Africa; 2 Division of Global HIV & TB, Global Health Center, U.S. Centers for Disease Control and Prevention, Nairobi, Kenya; 3 Division of Global HIV & TB, Global Health Center, U.S. Centers for Disease Control and Prevention, Atlanta, Georgia, United States of America; University of Cape Town, SOUTH AFRICA

## Abstract

The UNAIDS 95-95-95 targets are an important metric for guiding national HIV programs and measuring progress towards ending the HIV epidemic as a public health threat by 2030. Nevertheless, as proportional targets, the outcome of reaching the 95-95-95 targets will vary greatly across, and within, countries owing to the geographic diversity of the HIV epidemic. Countries and subnational units with a higher initial prevalence and number of people living with HIV (PLHIV) will remain with a larger number and higher prevalence of virally unsuppressed PLHIV—persons who may experience excess morbidity and mortality and can transmit the virus to others. Reliance on achievement of uniform proportional targets as a measure of program success can potentially mislead resource allocation and progress towards equitable epidemic control. More granular surveillance information on the HIV epidemic is required to effectively calibrate strategies and intensity of HIV programs across geographies and address current and projected health disparities that may undermine efforts to reach and sustain HIV epidemic control even after the 95 targets are achieved.

## Introduction

In 2014 the Joint United Nations Programme on HIV/AIDS (UNAIDS) rolled out the “Fast-Track” strategy to end the AIDS epidemic by 2030 [1, 2]. Since then, 144 countries adopted the ambitious Fast-Track treatment targets, aiming that by 2020, 90% of people living with HIV know their HIV status, 90% of those who know their status are on antiretroviral treatment (ART), and 90% of those on ART achieve viral load suppression (i.e. the 90-90-90 targets); and further, by 2025, aiming to achieve the even more ambitious 95-95-95 targets (95% of people living with HIV know their status, 95% with known status on ART, 95% on ART virally suppressed). The latter targets equate to 85.7% of all PLHIV being on ART and virally suppressed.

With an estimated 20.6 million people living with HIV (PLHIV), the 21 countries in Eastern and Southern Africa (ESA) accounted for over 80% of PLHIV on the continent, 54% of all PLHIV, and 45% (670,000) of new HIV infections worldwide in 2021 [3]. Overall, in 2021, 90% of PLHIV in the region knew their status, 78% of all PLHIV were on ART and 73% of all PLHIV had suppressed viral load [3]. Six ESA countries achieved the 90-90-90 targets by 2020, with several having also met, or made substantial progress towards, the second and third 95 targets and epidemic control [4]. As countries strive to reach or sustain the 95-95-95 targets with international aid decreasing [4] and national health budgets stretched to meet competing demands, addressing gaps in health equity and equality has emerged among the highest priorities of the global HIV response [4–6].

Whereas the need to address health inequity among specific populations (e.g., adolescent girls and young women, children, female sex workers, men who have sex with men, and persons who inject drugs) has featured strongly in global guidance for HIV programs, the need to address health inequities among geographically defined subpopulations with high burden of HIV has received less attention [6, 7]. We used published estimates of the number of adult (aged 15 years and above) PLHIV in the Eastern and Southern Africa region to assess the absolute number and population-prevalence of people living with HIV who are expected to remain virally unsuppressed (not on ART, or on ART but not virally suppressed [viral load >1,000 copies/ml]) regionally and nationally after reaching the 95-95-95 targets. We further explored the expected outcome of reaching the 95-95-95 targets at sub-national levels in two country case studies, Kenya, and South Africa.

## Methods

National and subnational data on the estimated number of PLHIV aged 15 years and above in 2022 were obtained from published UNAIDS modeled estimates [8, 9]. Population data on persons aged 15 years and above were obtained from published population estimates and projections [10] (S1 Table). We summed the overall number of PLHIV expected to be missed along each step of the 95-95-95 cascade to estimate the total number of virally unsuppressed PLHIV expected to remain in each country after meeting all three of the 95 targets. We then calculated the estimated prevalence of virally unsuppressed PLHV (viral load > 1,000 copies/ml); subsequently referred to as the “prevalence of virally unsuppressed PLHIV” expected to remain after reaching the 95-95-95 targets among the population of persons potentially susceptible to HIV infection. The population susceptible to HIV infection (i.e. HIV-negative population) was calculated by subtracting the total estimated number of PLHIV aged 15+ years from the total population aged 15+ years. We applied the same methodology to further calculate the number and prevalence of virally unsuppressed PLHIV at subnational levels in Kenya and South Africa using publicly available subnational HIV estimates and census data. Calculations were done using R version 4.3.0 [11]. Maps showing spatial variation in results among and within countries were generated using ArcGIS Enterprise version 10.6.1. This project was reviewed in accordance with CDC human research protection procedures and was determined to be non-research.

## Results

**Fig 1:**
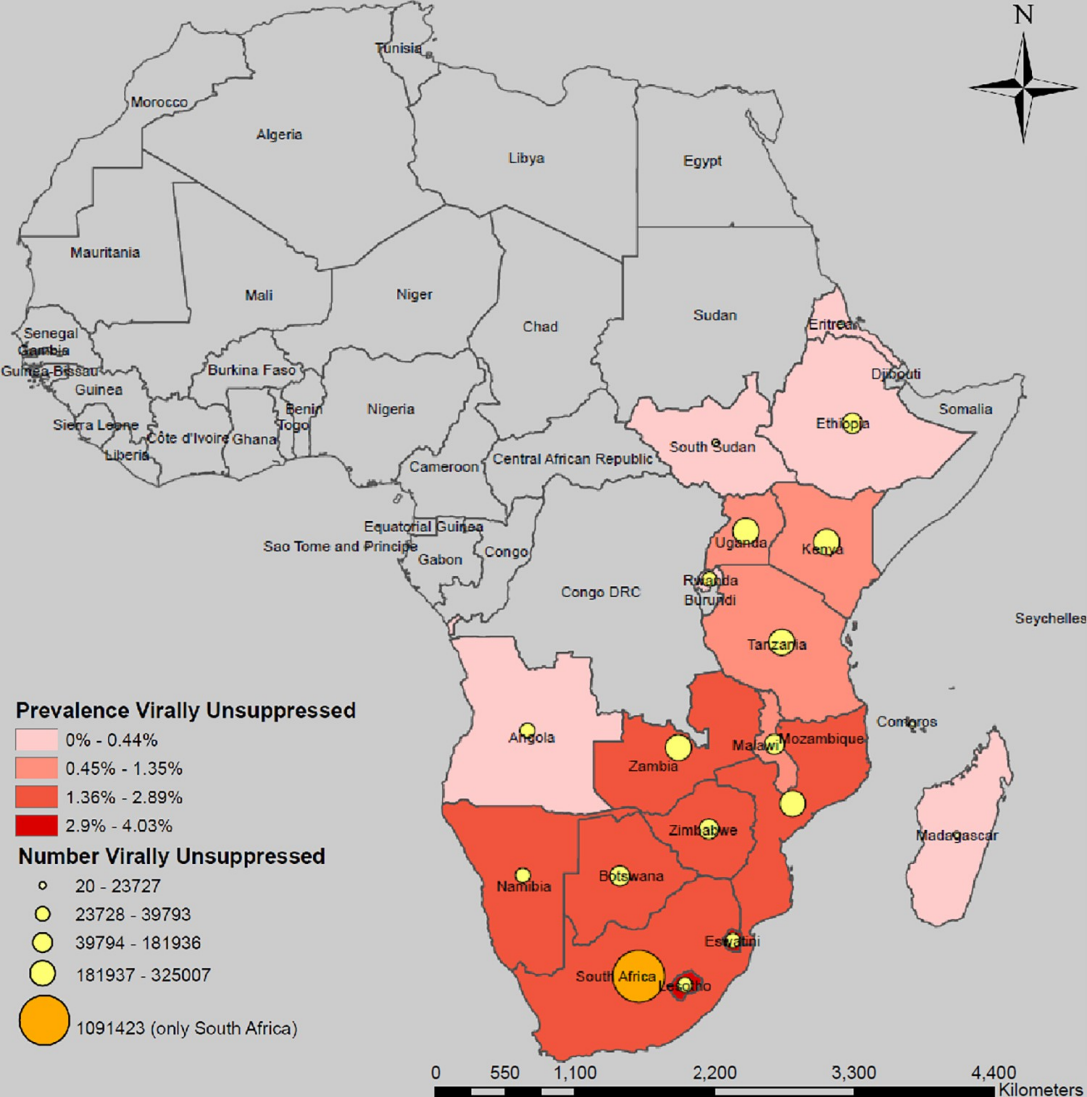
Estimated number and prevalence of virally unsuppressed people living with HIV aged 15+ years expected to remain after reaching the 95-95-95 targets by country, Eastern and Southern Africa Region. Maps were created using a licensed ArcGIS by ESRI version 10.6.1 GIS Mapping Software, Location Intelligence & Spatial Analytics | Esri; Base map from: Office of the Geographer and Global Issues, U.S. Department of State. https://catalog.data.gov/dataset/large-scale-international-boundaries; June 14, 2024.

Among the 21 countries included, 20 had national, and 15 had subnational estimates of PLHIV available. The number of virally unsuppressed PLHIV aged 15 years and above expected to remain after reaching all three 95 targets in the ESA region is 2,869,865 ranging from less than 2,000 in Comoros, Eritrea, and Mauritius to over 1.09 million in South Africa (Fig 1, Table 1). After reaching the 95-95-95 targets, eight countries are expected to remain with more than 100,000 virally unsuppressed PLHIV. The projected regional prevalence of virally unsuppressed PLHIV is 0.94%, and highest in Eswatini (4.03%), Lesotho (3.08%), South Africa (2.89%) and Botswana (2.87%).

**Table 1:** Estimated number and prevalence of virally unsuppressed people living with HIV aged 15+ years expected to remain after reaching the 95-95-95 targets by country, Eastern and Southern Africa Region.

			After reaching 1st 95	After reaching 2nd 95	After reaching 3rd 95	After reaching all 95-95-95 targets		
	Estimated number of PLHIV aged 15+ years	National prevalence of HIV, age 15+ years	Remain un-diagnosed	Know HIV status but not on ART	On ART but not virally suppressed	Total no. of virally non-suppressed PLHIV	Population denominator*	Prevalence virally non-suppressed PLHIV
Angola	278,549	1.42%	13,927	13,231	12,570	39,728	19,574,219	0.20%
Botswana	345,055	19.58%	17,253	16,390	15,571	49,213	1,713,277	2.87%
Comoros	140	0%	7	7	6	20	506,880	0.00%
Eritrea	12,000	0.60%	600	570	542	1,712	2,169,680	0.08%
Eswatini	216,083	27.17%	10,804	10,264	9,751	30,819	764,458	4.03%
Ethiopia	573,538	0.91%	28,677	27,243	25,881	81,801	63,266,546	0.13%
Kenya	1,309,914	4.14%	65,496	62,221	59,110	186,826	31,447,763	0.59%
Lesotho	266,871	20.98%	13,344	12,676	12,043	38,062	1,233,981	3.08%
Madagascar	57,000	0.30%	2,850	2,708	2,572	8,130	17,473,980	0.05%
Malawi	949,975	7.98%	47,499	45,124	42,868	135,490	11,769,331	1.15%
Mauritius	12,000	1.10%	600	570	542	1,712	1,072,680	0.16%
Mozambique	2,278,754	12.14%	113,938	108,241	102,829	325,007	18,450,997	1.76%
Namibia	208,155	12.62%	10,408	9,887	9,393	29,688	1,619,880	1.83%
Rwanda	228,772	2.80%	11,439	10,867	10,323	32,629	8,132,303	0.40%
South Africa	7,652,395	17.40%	382,620	363,489	345,314	1,091,423	37,755,782	2.89%
South Sudan	1,632,461	2.00%	8,318	7,902	7,507	23,727	7,132,561	0.33%
Tanzania	1,352,968	4.71%	81,623	77,542	73,665	232,830	34,462,677	0.68%
Uganda	1,344,900	5.51%	67,648	64,266	61,053	192,967	24,340,286	0.79%
Zambia	1,235,851	12.00%	67,245	63,883	60,689	191,816	11,020,044	1.74%
Zimbabwe	1,632,461	12.08%	61,793	58,703	55,768	176,263	10,050,707	1.75%
**Eastern and Southern Africa**	**19,955,382**	** **	**1,006,087**	**955,784**	**907,994**	**2,869,865**	**303,958,032**	**0.94%**

*Population denominator = Sum of total susceptible (HIV-negative) population aged 15+ years and the total number of PLHIV aged 15+ years who remain virally unsuppressed after reaching the 95-95-95 targets.

**Fig 2:**
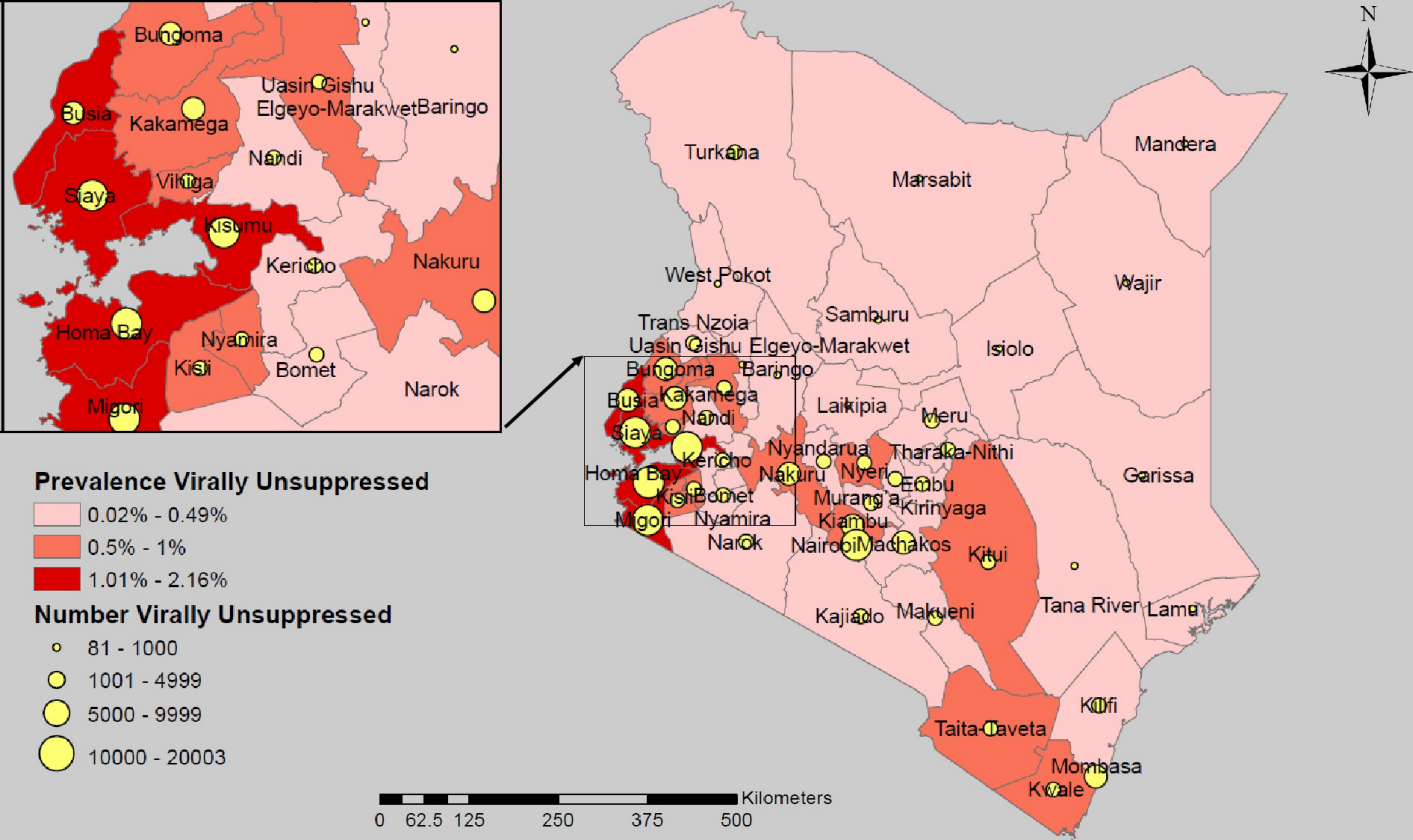
Estimated number and prevalence of virally unsuppressed people living with HIV aged 15+ years expected to remain after reaching the 95-95-95 targets by subnational unit, Kenya. Maps were created using a licensed ArcGIS by ESRI version 10.6.1 GIS Mapping Software, Location Intelligence & Spatial Analytics | Esri, Desktop Help 10.0 - Redistribution rights (arcgis.com); Base map from: Esri. "Kenya_4_Counties_2022_Nov”[basemap]. Kenya_4_Counties_2022_Nov (FeatureServer)(arcgis.com); (June 14, 2024).

The implications of national achievement of the 95 targets differ greatly at subnational level. In Kenya, the projected outcome of meeting the 95-95-95 targets nationally leaves 186,826 virally unsuppressed PLHIV, yielding a national prevalence of virally unsuppressed PLHIV of 0.59% (Fig 2, Table 2). Approximately 50% (91,359) of the remaining unsuppressed PLHIV reside in 7 of the 47 subnational units (counties): Homa Bay, Kisumu, Siaya, Migori, Mombasa, Nakuru and Nairobi. The prevalence of unsuppressed PLHIV is expected to be 2-fold higher than the regional estimate for ESA, and over 3-fold higher than the Kenya national estimate in four counties in western Kenya around Lake Victoria, namely, Kisumu (2.16%), Homa Bay (2.15%), Migori (2.03%) and Siaya (1.92%).

**Table 2:** Estimated number and prevalence of virally unsuppressed people living with HIV aged 15+ years expected to remain after reaching the 95-95-95 targets by subnational unit, Kenya.

			After reaching	After reaching	After reaching	After reaching		
			1st 95	2nd 95	3rd 95	all 95-95-95 targets		
Subnational Unit	Estimated number of PLHIV aged 15+ years	HIV prevalence (%)	Remain undiagnosed	Know HIV status, not on ART	On ART, not virally suppressed	Total no. of virally non-suppressed PLHIV	Population denominator*	Prevalence virally non-suppressed PLHIV
**Central**	**119,495 **	**2.99% **	**5,975 **	**5,676 **	**5,392 **	**17,043 **	**3,977,974 **	**0.43% **
Kiambu	65,438	3.62%	3,272	3,108	2,953	9,333	1,800,642	0.52%
Kirinyaga	11,586	2.45%	579	550	523	1,652	471,689	0.35%
Murang’a	17,723	2.28%	886	842	800	2,528	776,495	0.33%
Nyandarua	7,386	1.65%	369	351	333	1,053	445,668	0.24%
Nyeri	17,362	3.57%	868	825	783	2,476	483,478	0.51%
**Coast **	**113,196 **	**4.01% **	**5,660 **	**5,377 **	**5,108 **	**16,145 **	**2,805,399 **	**0.58% **
Kilifi	21,735	2.40%	1,087	1,032	981	3,100	903,425	0.34%
Kwale	23,085	4.44%	1,154	1,097	1,042	3,292	516,215	0.64%
Lamu	1,731	1.82%	87	82	78	247	94,854	0.26%
Mombasa	56,559	6.42%	2,828	2,687	2,552	8,067	872,960	0.92%
Taita-Taveta	8,420	3.47%	421	400	380	1,201	241,526	0.50%
Tana River	1,667	0.94%	83	79	75	238	176,418	0.13%
**Eastern **	**130,880 **	**2.73% **	**6,544 **	**6,217 **	**5,906 **	**18,667 **	**4,776,826 **	**0.39% **
Embu	10,184	2.22%	509	484	460	1,453	457,188	0.32%
Isiolo	1,991	1.24%	100	95	90	284	160,356	0.18%
Kitui	29,958	3.98%	1,498	1,423	1,352	4,273	748,288	0.57%
Machakos	35,224	3.33%	1,761	1,673	1,589	5,024	1,053,961	0.48%
Makueni	19,266	2.78%	963	915	869	2,748	690,362	0.40%
Marsabit	1,340	0.49%	67	64	60	191	274,163	0.07%
Meru	25,723	2.31%	1,286	1,222	1,161	3,669	1,108,721	0.33%
Tharaka	7,194	2.53%	360	342	325	1,026	283,787	0.36%
Nithi								
**Nairobi**	**140,251 **	**4.44% **	**7,013 **	**6,662 **	**6,329 **	**20,003 **	**3,137,702 **	**0.64% **
**North Eastern**	**3,029 **	**0.21% **	**151 **	**144 **	**137 **	**432 **	**1,466,100 **	**0.03% **
Garissa	1,480	0.23%	74	70	67	211	648,345	0.03%
Mandera	981	0.24%	49	47	44	140	411,669	0.03%
Wajir	568	0.14%	28	27	26	81	406,087	0.02%
**Nyanza **	**432,991 **	**11.00% **	**21,650 **	**20,567 **	**19,539 **	**61,755 **	**3,875,668 **	**1.59% **
Homa Bay	100,073	14.76%	5,004	4,753	4,516	14,273	663,638	2.15%
Kisii	30,346	3.73%	1,517	1,441	1,369	4,328	808,314	0.54%
Kisumu	113,236	14.79%	5,662	5,379	5,110	16,150	749,349	2.16%
Migori	92,275	13.94%	4,614	4,383	4,164	13,161	648,782	2.03%
Nyamira	14,385	3.64%	719	683	649	2,052	392,680	0.52%
Siaya	82,676	13.23%	4,134	3,927	3,731	11,792	612,904	1.92%
**Rift Valley **	**214,541 **	**2.59% **	**10,727 **	**10,191 **	**9,681 **	**30,599 **	**8,239,727 **	**0.37% **
Baringo	4,668	1.14%	233	222	211	666	407,707	0.16%
Bomet	10,019	1.79%	501	476	452	1,429	559,395	0.26%
Elgeyo-	5,781	1.99%	289	275	261	825	290,045	0.28%
Marakwet								
Kajiado	23,829	3.10%	1,191	1,132	1,075	3,399	764,464	0.44%
Kericho	17,503	2.89%	875	831	790	2,496	602,715	0.41%
Laikipia	6,309	1.75%	315	300	285	900	358,585	0.25%
Nakuru	55,481	3.70%	2,774	2,635	2,504	7,913	1,490,503	0.53%
Nandi	14,073	2.39%	704	668	635	2,007	587,718	0.34%
Narok	12,157	1.85%	608	577	549	1,734	655,696	0.26%
Samburu	3,329	1.90%	166	158	150	475	175,002	0.27%
Trans Nzoia	14,588	2.32%	729	693	658	2,081	626,085	0.33%
Turkana	12,099	2.12%	605	575	546	1,726	568,278	0.30%
Uasin Gishu	32,320	3.95%	1,616	1,535	1,458	4,610	813,889	0.57%
West Pokot	2,384	0.70%	119	113	108	340	339,643	0.10%
**Western **	**155,531 **	**4.87% **	**7,777 **	**7,388 **	**7,018 **	**22,183 **	**3,168,367 **	**0.70% **
Bungoma	43,303	4.15%	2,165	2,057	1,954	6,176	1,037,912	0.60%
Busia	41,182	7.23%	2,059	1,956	1,858	5,874	564,040	1.04%
Kakamega	51,645	4.33%	2,582	2,453	2,330	7,366	1,185,615	0.62%
Vihiga	19,400	5.06%	970	921	875	2,767	380,800	0.73%
**Kenya **	**1,309,914 **	**4.14% **	**65,496 **	**62,221 **	**59,110 **	**186,826 **	**31,447,763 **	**0.59% **

*Population denominator = Sum of the total susceptible (HIV-negative) population aged 15+ years and the total number of PLHIV aged 15+ years who remain virally unsuppressed after reaching the 95-95-95 targets.

**Fig 3:**
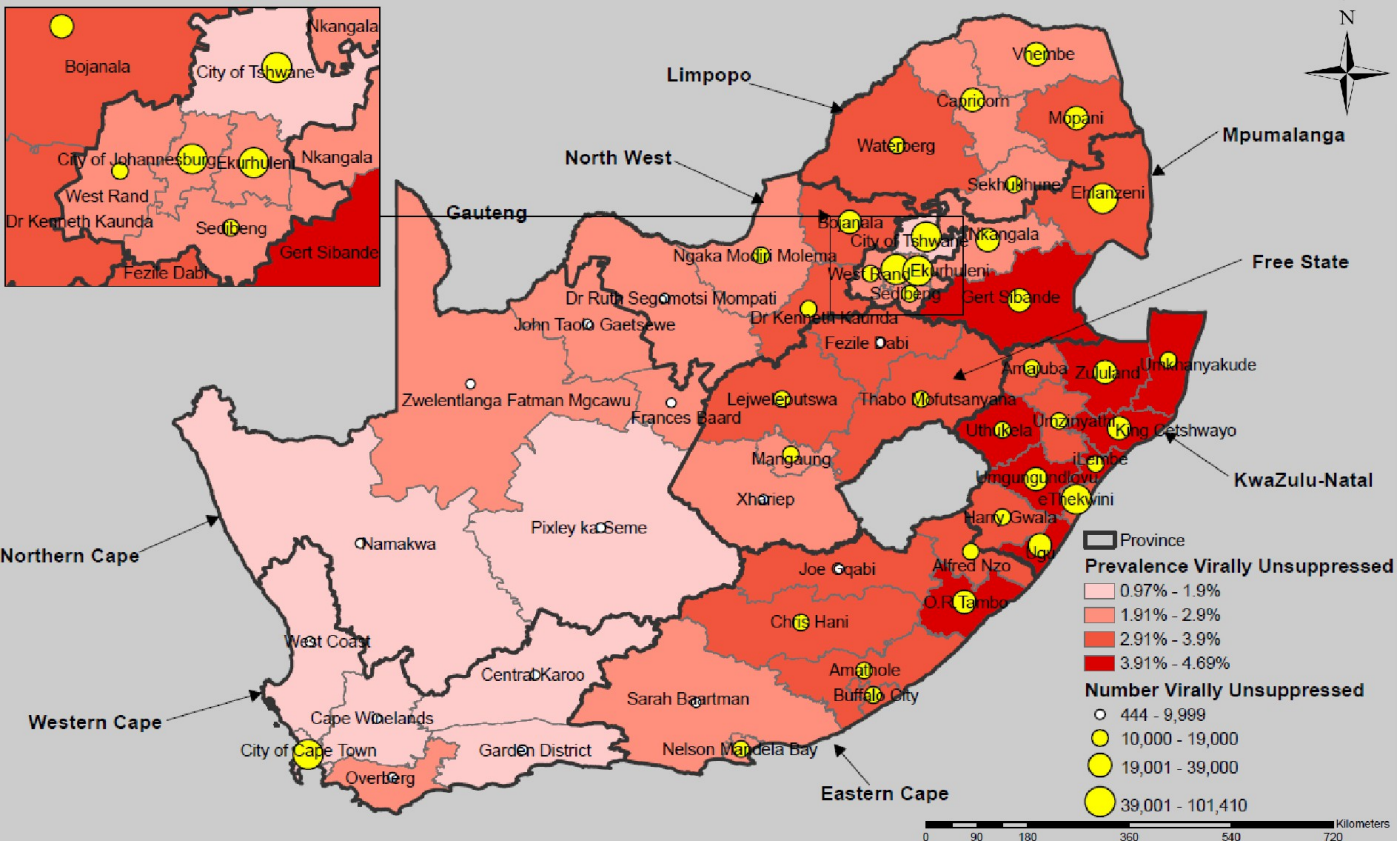
Estimated number and prevalence of virally unsuppressed people living with HIV aged 15+ years expected to remain after reaching the 95-95-95 targets by subnational unit, South Africa. Maps were created using a licensed ArcGIS by ESRI version 10.6.1 GIS Mapping Software, Location Intelligence & Spatial Analytics | Esri, Desktop Help 10.0 - Redistribution rights (arcgis.com); Base map from: Esri. “SouthAfrica_5_Districts_2022_Nov”[basemap]. SouthAfrica_5_Districts_2022_Nov (FeatureServer) (arcgis.com); (June 14, 2024).

In South Africa the projected outcome of meeting the 95-95-95 targets leaves 1,091,423 virally unsuppressed PLHIV, translating to a national prevalence of unsuppressed PLHIV of 2.89%. Outcomes vary widely across the nine provinces and 52 municipalities (districts) (Fig 3, Table 3). Fifteen (29%) districts remain with fewer than 10,000 virally unsuppressed PLHIV, 31 (60%) with 10,000–39,000, and 6 (11%) with over 40,000. Nearly one-half (26 districts) have a projected prevalence of virally unsuppressed PLHIV of 3.0% and above—≥ 3-fold higher than the regional estimate for ESA—with highest expected prevalence in districts in KwaZulu Natal Province (range 3.48%–4.69%).

**Table 3:** Estimated number and prevalence of virally unsuppressed people living with HIV aged 15+ years expected to remain after reaching the 95-95-95 targets by subnational unit, South Africa.

			After reaching 1st 95	After reaching 2nd 95	After reaching 3rd 95	After reaching all 95-95-95 targets		
Subnational Unit	Estimated no. PLHIV aged 15+ years	HIV prevalence (%)	Remain undiagnosed	Know HIV status, not on ART	On ART, not virally suppressed	Total no. virally non-suppressed PLHIV	Population denominator*	Prevalence virally non-suppressed PLHIV
Eastern Cape Province	868,185	18.5%	43,409	41,239	39,177	123,825	4,027,238	3.07%
Alfred Nzo District	105,157	20.7%	5,258	4,995	4,745	14,998	436,250	3.44%
Amathole District	95,046	17.9%	4,752	4,515	4,289	13,556	456,053	2.97%
Buffalo City Metropolitan	120,808	19.5%	6,040	5,738	5,451	17,230	531,907	3.24%
Chris Hani District	90,656	19.0%	4,533	4,306	4,091	12,930	409,713	3.16%
Oliver Tambo District	232,589	23.6%	11,629	11,048	10,496	33,173	847,326	3.92%
Joe Gqabi District	42,317	18.0%	2,116	2,010	1,910	6,035	197,755	3.05%
Nelson Mandela Bay	126,821	18.4%	6,341	6,024	5,723	18,088	829,236	2.18%
Sarah Baartman District	54,791	14.8%	2,740	2,603	2,472	7,815	318,998	2.45%
** Free State Province**	**400,276**	**19.4%**	**20,014**	**19,013**	**18,062**	**57,089**	**1,778,361**	**3.21%**
Lejweleputswa District	94,784	20.1%	4,739	4,502	4,277	13,519	404,948	3.34%
Thabo Mofutsanyane	113,022	21.8%	5,651	5,369	5,100	16,120	445,035	3.62%
District								
Fezile Dabi District	69,696	19.2%	3,485	3,311	3,145	9,940	312,891	3.18%
Mangaung Metropolitan	109,947	17.4%	5,497	5,222	4,961	15,681	541,877	2.89%
Xhariep District	12,827	15.0%	641	609	579	1,829	73,611	2.48%
** Gauteng Province**	**1,800,724**	**14.4%**	**90,036**	**85,534**	**81,258**	**256,828**	**10,726,635**	**2.39%**
City of Johannesburg	711,023	14.7%	35,551	33,774	32,085	101,410	4,159,706	2.44%
Metropolitan								
City of Tshwane	344,530	11.5%	17,227	16,365	15,547	49,139	2,582,677	1.90%
Metropolitan								
Ekurhuleni Metropolitan	530,480	16.8%	26,524	25,198	23,938	75,660	2,712,327	2.79%
Sedibeng District	731,014	14.2%	5,176	4,918	4,672	14,766	628,672	2.35%
West Rand District	747,968	14.9%	5,558	5,280	5,016	15,855	643,252	2.46%
** KwaZulu Natal Province**	**1,949,499**	**24.1%**	**97,475**	**92,601**	**87,971**	**278,047**	**6,953,144**	**4.00%**
eThekwini Metropolitan	645,186	21.0%	32,259	30,646	29,114	92,020	2,647,386	3.48%
Harry Gwala District	75,117	22.9%	3,756	3,568	3,390	10,714	281,642	3.80%
King Cetshwayo District	180,842	28.3%	9,042	8,590	8,160	25,793	550,502	4.69%
Ugu District	140,644	26.0%	7,032	6,681	6,347	20,059	464,323	4.32%
uMgungundlovu District	229,654	27.7%	11,483	10,909	10,363	32,754	714,067	4.59%
Uthukela District	119,695	26.1%	5,985	5,686	5,401	17,071	393,857	4.33%
Zululand District	147,574	26.7%	7,379	7,010	6,659	21,048	475,934	4.42%
Amajuba District	90,340	23.0%	4,517	4,291	4,077	12,885	337,871	3.81%
iLembe District	124,487	25.9%	6,224	5,913	5,617	17,755	412,661	4.30%
Umkhanyakude District	117,662	27.2%	5,883	5,589	5,309	16,782	371,923	4.51%
Umzinyathi District	78,298	22.2%	3,915	3,719	3,533	11,167	302,977	3.69%
** Limpopo Province**	**682,578**	**17.2%**	**34,129**	**32,422**	**30,801**	**97,353**	**3,406,584**	**2.86%**
Capricorn District	150,837	17.1%	7,542	7,165	6,807	21,513	760,696	2.83%
Mopani District	161,120	20.2%	8,056	7,653	7,271	22,980	687,588	3.34%
Sekhukhune District	112,749	14.5%	5,637	5,356	5,088	16,081	666,659	2.41%
Vhembe District	153,859	15.8%	7,693	7,308	6,943	21,944	835,664	2.63%
Waterberg District	104,013	19.6%	5,201	4,941	4,694	14,835	455,977	3.25%
** Mpumalanga Province**	**735,931**	**20.9%**	**36,797**	**34,957**	**33,209**	**104,962**	**3,021,013**	**3.47%**
Ehlanzeni District	300,316	23.2%	15,016	14,265	13,552	42,833	1,114,169	3.84%
Gert Sibande District	235,240	24.8%	11,762	11,174	10,615	33,551	817,366	4.10%
Nkangala District	200,375	15.8%	10,019	9,518	9,042	28,578	1,089,478	2.62%
** Northern Cape Province**	**107,485**	**13.8%**	**5,374**	**5,106**	**4,850**	**15,330**	**667,472**	**2.30%**
Frances Baard District	41,433	16.9%	2,072	1,968	1,870	5,909	210,586	2.81%
John Taolo Gaetsewe	27,044	17.2%	1,352	1,285	1,220	3,857	135,327	2.85%
District								
Namakwa District	4,732	6.6%	237	225	214	675	61,637	1.10%
Pixley ka Seme District	13,297	10.5%	665	632	600	1,896	108,798	1.74%
Zwelentlanga Fatman	20,979	11.9%	1,049	997	947	2,992	151,124	1.98%
Mgcawu District								
** North West Province**	**524,486**	**17.8%**	**26,224**	**24,913**	**23,667**	**74,805**	**2,534,153**	**2.95%**
Bojanala Platinum District	268,067	18.5%	13,403	12,733	12,097	38,233	1,247,310	3.07%
Dr Kenneth Kaunda	109,260	19.0%	5,463	5,190	4,930	15,583	494,766	3.15%
District								
Ngaka Modiri Molema	99,852	16.2%	4,993	4,743	4,506	14,241	531,105	2.68%
District								
Dr Ruth Segomotsi	47,307	15.6%	2,365	2,247	2,135	6,747	260,973	2.59%
Mompati District								
** Western Cape Province**	**583,231**	**10.8%**	**29,162**	**27,704**	**26,318**	**83,183**	**4,641,182**	**1.79%**
City of Cape Town	399,320	11.1%	19,966	18,968	18,019	56,953	3,089,927	1.84%
Metropolitan								
Cape Winelands District	66,742	9.3%	3,337	3,170	3,012	9,519	614,897	1.55%
Central Karoo District	3,115	5.9%	156	148	141	444	45,605	0.97%
Garden Route District	49,476	10.7%	2,474	2,350	2,233	7,057	398,689	1.77%
Overberg District	27,016	11.9%	1,351	1,283	1,219	3,853	195,586	1.97%
West Coast District	37,562	10.9%	1,878	1,784	1,695	5,357	296,478	1.81%
**South Africa Overall**	**7,652,395**	**17.4%**	**382,620**	**363,489**	**345,314**	**1,091,423**	**37,755,782**	**2.89%**

*Population denominator = Sum of total susceptible (HIV-negative) population aged 15+ years and the total number of PLHIV aged 15+ years who remain virally unsuppressed after reaching the 95-95-95 targets.

## Discussion

Our analysis demonstrates, that although the UNAIDS 95-95-95 targets are an important metric for guiding and monitoring national HIV programs, the application of uniform proportional targets across the geographically heterogeneous HIV epidemic in ESA fails to fully address health inequities. Using HIV estimates for 2022 we show that if all countries had reached all three of the UNAIDS 95-95-95 targets, those with a higher initial HIV prevalence and number of PLHIV would remain with a greater number and prevalence of virally unsuppressed PLHIV—essentially, a greater number and prevalence of PLHIV who, without treatment, may have poor health outcomes and can transmit the virus. We further demonstrate that these limitations, and corresponding concerns about equitable epidemic control, persist when applied across subnational units in the case studies of Kenya and South Africa.

Kenya, with approximately 1,310,000 million PLHIV aged 15+ years, had a UNAIDS target achievement of 96-89-94 in 2021 [4]. With annual HIV incidence of 1.17 per 1,000 adults aged 15–49 years, and estimated new HIV infections (~35,000) falling below deaths among PLHIV (~36,000) [4], Kenya is among numerous countries in ESA nearing both the 95-95-95 targets and widely used definitions of HIV epidemic control [4, 12]. Our analysis highlights that despite these promising national metrics, geographic disparities in the remaining burden of HIV will persist at county-level, particularly in western Kenya around Lake Victoria. These counties will need to achieve a population-level viral load suppression that *exceeds* 85.7% (i.e., exceeds the 95-95-95 targets) to reach a prevalence of unsuppressed PLHIV equivalent to the national projection. For example, Kisumu County, would need approximately 96% (108,706) of its 113,236 PLHIV to be virally suppressed—nearly a 99-99-99 target achievement—to reach the current projected national prevalence of 0.59%.

In South Africa, with an estimated 7.5 million PLHIV, 94% are aware of their HIV status; however, gaps remain in treatment uptake among those who know their status (76%), and to a lesser degree, viral load suppression among those on ART (92%). Nationally, the number of new HIV infections in 2021 was 210,000, corresponding to an HIV incidence of 6.9 per 1,000 adults aged 15–49 years [3]. Owing to the sheer magnitude of the HIV epidemic, the expected number and prevalence of virally unsuppressed PLHIV after reaching the 95-95-95 targets in South Africa far exceeds the projected remaining burden for other countries in the region. Overall, 94.4% (7,224,438/7,652,395) of South Africa’s PLHIV would need to be virally suppressed, a 98-98-98 national target achievement, to reach a prevalence of unsuppressed PLHIV equivalent to the ESA regional projection (1.03%). Differences are even more pronounced at subnational levels, where, after reaching the 95s (85.7% population VL suppression) some provinces (KwaZulu-Natal, Gauteng) are expected to remain with a larger number and prevalence of virally unsuppressed PLHIV than some countries had nationally at *baseline* before applying the 95-95-95 target cascade.

Both case studies underscore the value of subnational estimates of PLHIV [9] and need for a more granular approach to defining and assessing progress towards ending HIV as a public health threat within and across geographically diverse HIV epidemics. In areas with generalized HIV epidemics, such as the ESA, the impact of achieving the 95-95-95 treatment targets by 2030, and recently expanded set of HIV prevention targets by 2025, is estimated using the Goals Age-Structured Model (Goals-ASM), described elsewhere [13]. Briefly, the Goals-ASM model incorporates data on behaviors, epidemiological factors, and biomedical and behavioral interventions that can influence the probability of HIV transmission, together with data on HIV prevalence, key populations size estimates and intervention coverage, all stratified by age and sex, to generate estimates of expected trends in new infections and AIDS-related deaths. Indicators are generated at national level and aggregated up to produce a global impact estimate, which is validated against results from other models [13, 14].

Whereas the Goals-ASM model of the global HIV epidemic focuses on national and regional outcomes, our analysis points to a critical need for models that assess the prevention and treatment intervention coverage required at the subnational level to effectively, and equitably, reduce the number and prevalence of virally unsuppressed PLHIV, new HIV infections and deaths across a geographically diverse epidemic. Finer detail could assist national HIV programs to effectively calibrate strategies and the intensity of programing across geographic areas, and to address current and projected health disparities that may undermine efforts to reach and sustain HIV epidemic control even after the 95 targets are achieved.

Population-level surveys are key sources of data on incidence and prevalence of HIV, knowledge of HIV status, prevalence of viral load non-suppression in the population of PLHIV, and prevalence of behavioral factors that can affect the risk of HIV transmission—these data are essential for monitoring program impact and gaps, and as a source of national and subnational HIV model inputs and assumptions [9, 15, 16]. Numerous countries in ESA region have supported one or more periodic (approximately every 5 years) national population-based HIV serological and behavioral surveys in the last 10 years, in some cases oversampling geographic areas with high burden of HIV to provide subnational estimates of key HIV indicators [17–19]. The relative infrequency and cost of national surveys in an era of a rapidly evolving HIV response limits timely access to valuable data for decision-making, an issue that could be addressed by more frequent population surveys economized to focus on geographic areas and subpopulations with greatest burden of HIV and/or greatest potential barriers to accessing care [18]. HIV case surveillance systems provide ongoing longitudinal data on key outcomes (HIV diagnosis, viral load status, mortality) among persons with HIV infection who have accessed care. Case surveillance data can be used to monitor HIV epidemics, inform HIV programing and guide rapid public health action at a granular level [20]; however, the status of implementation of case surveillance, including collection of mortality events, varies widely across countries [21]. Expansion of effective HIV case surveillance systems [21], including linkage to high quality vital registration data [22], together with more frequent localized population surveys could improve the availability of timely, granular data needed to guide HIV programing, effectively track, model, and control the HIV epidemic, and address existing and emergent inequities at subnational levels.

### Limitations

Our analysis has limitations. Firstly, we used census projections for 2018–2021; any differences between projected and actual population aged 15 years could result in an over- or under-estimation of the calculated prevalence of virally unsuppressed PLHIV. Secondly, this analysis relied on modeled estimates of the number of PLHIV. Published HIV estimates and associated credible intervals are generated using a robust, standardized process [9], but are nevertheless impacted by the quality and timeliness of model inputs for each country. Finally, as our analysis applied the 95-95-95 targets to current national and subnational estimates targets to calculate projected outcomes, it did not account for longitudinal changes in population structure, transmission dynamics, migration [23, 24] or the public health response (e.g. expanded access to pre-exposure prophylaxis), which over time could impact the course of the HIV epidemic in the general population, key and priority populations (i.e., female sex workers, men who have sex with men, people who inject drugs, adolescent girls and young women). Models tailored to the local (geographic) context would lend further insight into how these may impact projected outcomes. Despite these limitations, the underlying principle that health inequities result when a uniform set of targets is applied across a heterogeneous HIV epidemic remains unchanged.

## Conclusions

The UNAIDS 95-95-95 targets are an important metric for guiding and monitoring national HIV programs. Our analysis demonstrates that reliance on uniform targets across a geographically diverse HIV epidemic can lead to remarkably different outcomes, and potentially mislead program strategies, resource allocation, and progress towards equitable epidemic control. More granular surveillance information on the HIV epidemic could assist national HIV programs to effectively calibrate strategies and intensity of programing across geographic areas to address current and projected health disparities that may undermine efforts to reach and sustain HIV epidemic control even after the 95 targets are achieved.

## Supporting information

S1 Table Data sources and available data. Description of data sources and available data for calculation of the number and prevalence of virally unsuppressed people living with HIV by country.
(DOCX)
