# Supplementary material for: Beyond the 95s: What happens when uniform program targets are applied across a heterogenous HIV epidemic in Eastern and Southern Africa?
Source: PLOS Glob Public Health. 2024 Sep 19;4(9):e0003723. doi: 10.1371/journal.pgph.0003723 (PMC11412679; doi:10.1371/journal.pgph.0003723)
Supplement: S1 Table — Description of data sources and available data for calculation of the number and prevalence of virally unsuppressed people living with HIV by country. (DOCX) [file pgph.0003723.s001.docx]

|  | Data on estimated number of PLHIV available (8) | | Actual or projected census data (Year*) (10) |
| --- | --- | --- | --- |
| **Country** | **National** | **Sub-national** |  |
| Angola | Yes | Yes | Projected (2022) |
| Botswana | Yes | Yes | Projected (2020) |
| Comoros | Yes | No | Projected (2021) |
| Eritrea | Yes | No | Projected (2021) |
| Eswatini | Yes | Yes | Actual (2021) |
| Ethiopia | Yes | Yes | Projected (2022) |
| Kenya | Yes | Yes | Projected (2022) |
| Lesotho | Yes | Yes | Projected (2020) |
| Madagascar | Yes | No | Projected (2021) |
| Malawi | Yes | Yes | Projected (2021) |
| Mauritius | Yes | No | Projected (2021) |
| Mozambique | Yes | Yes | Projected (2022) |
| Namibia | Yes | Yes | Projected (2011) |
| Rwanda | Yes | Yes | Projected (2021) |
| Seychelles | No | No | Not Applicable |
| South Africa | Yes | Yes | Projected (2020) |
| South Sudan | Yes | Yes | Projected (2008) |
| Tanzania | Yes | Yes | Projected (2018) |
| Uganda | Yes | Yes | Projected (2021) |
| Zambia | Yes | Yes | Projected (2021) |
| Zimbabwe | Yes | Yes | Projected (2020) |

*Year in which the actual population census occurred, or year to which
population census data were projected.
